# DraculR: A Web-Based Application for In Silico Haemolysis Detection in High-Throughput microRNA Sequencing Data

**DOI:** 10.3390/genes14020448

**Published:** 2023-02-09

**Authors:** Melanie D. Smith, Shalem Y. Leemaqz, Tanja Jankovic-Karasoulos, Dylan McCullough, Dale McAninch, Anya L. Arthurs, James Breen, Claire T. Roberts, Katherine A. Pillman

**Affiliations:** 1 Flinders Health and Medical Research Institute, Flinders University, Bedford Park, SA 5042, Australia; 2 Adelaide Medical School, University of Adelaide, Adelaide, SA 5005, Australia; 3 Indigenous Genomics, Telethon Kids Institute, Adelaide, SA 5000, Australia; 4 College of Health & Medicine, Australian National University, Canberra, ACT 2600, Australia; 5 Centre for Cancer Biology, an Alliance between SA Pathology and the University of South Australia, Adelaide, SA 5000, Australia; 6 School of Biological Sciences, University of Adelaide, Adelaide, SA 5005, Australia

**Keywords:** haemolysis, microRNA, plasma, biomarker, prediction, bioinformatics

## Abstract

The search for novel microRNA (miRNA) biomarkers in plasma is hampered by haemolysis, the lysis and subsequent release of red blood cell contents, including miRNAs, into surrounding fluid. The biomarker potential of miRNAs comes in part from their multicompartment origin and the long-lived nature of miRNA transcripts in plasma, giving researchers a functional window for tissues that are otherwise difficult or disadvantageous to sample. The inclusion of red-blood-cell-derived miRNA transcripts in downstream analysis introduces a source of error that is difficult to identify posthoc and may lead to spurious results. Where access to a physical specimen is not possible, our tool will provide an in silico approach to haemolysis prediction. We present DraculR, an interactive Shiny/R application that enables a user to upload miRNA expression data from a short-read sequencing of human plasma as a raw read counts table and interactively calculate a metric that indicates the degree of haemolysis contamination. The code, DraculR web tool and its tutorial are freely available as detailed herein.

## 1. Introduction

Circulating miRNAs have long been identified in human plasma and, given their stability in this medium, have strong potential as biomarkers. While there are multiple techniques for quantifying the abundance of miRNAs in plasma, high-throughput sequencing detects both known and novel (i.e., putative) miRNAs with single-base resolution. The fine resolution provided by high-throughput sequencing allows distinction between variants differing by a single nucleotide, as well as isomiRs of differing lengths [1] with many researchers now leveraging this technology to identify and quantify the abundance of plasma miRNAs [2,3,4,5,6,7,8,9,10,11]. The biomarker potential of plasma miRNAs is in part because plasma-derived transcripts commonly originate from varied endogenous compartments. The accurate profiling of plasma miRNAs is hindered when transcripts are derived from another blood source such as red blood cells. When haemolysis occurs due to the shearing of red blood cells during blood sampling, miRNAs are released into the volume of blood drawn [12,13,14,15,16]. The presence of red-blood-cell-associated miRNAs alters the plasma expression profile, affecting the global normalisation of sequence counts [1].

The increase in the relative abundance of red-blood-cell-associated miRNAs and the aberrant normalisation of libraries have potential to impact the profile analysis of miRNAs [12,13,15,16], yet an assessment of haemolysis is rarely reported. As part of a suite of data quality checks and controls prior to the analysis of miRNA high-throughput sequencing data [17,18], one should include an assessment of haemolysis in the plasma sample from which the sequencing library was produced. Recently, we developed a method that uses a data-driven approach for the assessment of haemolysis confounding in silico [19]. This method does not require access to the original plasma sample, an advantage over both of the two long-used gold standard approaches. The first of these approaches, the delta quantification cycle (ΔCq), uses expression levels of a known blood-cell-associated miRNA (miR-451a) and a control miRNA (miR-23a-3p) to determine the difference between the two raw Cq values [12]. The second uses a Spectrophotometry approach based on the absorbance maximum of free haemoglobin measured at 414 nm [20]. In this work, we present DraculR, an easy-to-use computational tool that enables the user to upload self-generated or publicly available miRNA high-throughput sequencing expression data for assessments and returns both visual and tabular recommendations for downstream analysis.

## 2. Materials and Methods

DraculR is an interactive Shiny/R web-based tool for the in silico assessment of haemolysis contributions to small RNA sequencing libraries prepared from human plasma. DraculR calculates and provides visualisations of our previously described Haemolysis Metric [19], which is analogous to the gold-standard quantitative PCR-based ΔCq (miR-23a-3p–miR-451a) method that determines the difference between the abundance of two miRNAs, one known to vary and one known to be invariant in the presence of haemolysis. A full description and validation of the Haemolysis Metric has been detailed previously [19]. Briefly, the Haemolysis Metric is calculated as the sample-specific difference in geometric means of the normalised gene expression values between two sets of miRNAs: firstly, 20 miRNAs identified as indicative of haemolysis (‘signature set’), and secondly, all other miRNAs (‘background’). This set of twenty was designed to capture a set of reliably quantifiable (i.e., abundant) miRNAs that are highly overrepresented in red blood cells with enough redundancy that the removal of a small number of user-defined miRNAs should not excessively compromise the accuracy of the measure.

Prior to the calculation of the Haemolysis Metric, the user has the opportunity to manually choose miRNAs to exclude from the signature and background sets. Using this feature, the user should exclude all miRNAs from the signature set whose differential expression correlates to the biology of the dataset, although others can also be excluded if desired. Then, the geometric mean of the reduced signature set will be calculated, as defined in (1). Let $Z_{x}$ be the miRNA reduced signature set (log_2_ counts per million counts) and $Z_{y}$ be the background miRNA set (log_2_ counts per million counts), where $x=1,2,3,\ldots ,p_{1}$ with p_1_ = the number of miRNAs in the reduced signature set and $y=1,2,3,\ldots ,p_{2}$ where p_2_ = the number of miRNAs in the background and i=1,2,3,…,n where *n* = the sample size after filtering:

(1) $$Haemolysis\;Metric=\sqrt[{p_{1}}]{\overset{p_{1}}{\underset{x=1}{\prod }}Z_{x_{i}}}-\sqrt[{p_{2}}]{\overset{p_{2}}{\underset{y=1}{\prod }}Z_{y}}\;$$

## 3. Results

### 3.1. Application

**Figure 1:**
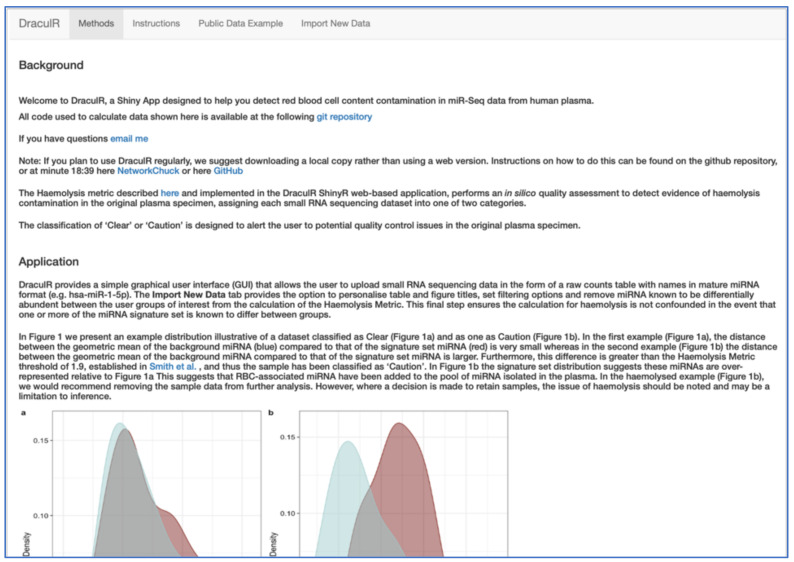
Screenshot of the DraculR Shiny Application main page. Navigation of the DraculR application is achieved by using the four tabs (“Methods”, “Instructions”, “Public Data Example”, “Import New Data”) located at the top of the page.

DraculR is written in the R/Shiny web development framework [21] and can either be run online (https://mxhp75.shinyapps.io/DraculR) or downloaded from our GitHub repository (https://github.com/mxhp75/DraculR) and run locally. The instructions for setting up a local copy of DraculR can be found on the repository website. DraculR is structured to comprise four tabs, accessible from the front page (Figure 1). The main page and first tab (‘Methods’) present a detailed description of the Haemolysis Metric method which underpins the approach. The ‘Instructions’ tab details how to use the app and the formatting requirements for the input file. The ‘Public Data Example’ allows the user to explore example results and graphics for each of the four publicly available datasets, and the ‘Import New Data’ tab may be used for performing analyses on user-generated datasets. For more information detailing the analyses of public data, see the Supplementary Materials Figure S1 which details the instruction for user imported data.

When red-blood-cell-associated miRNA transcripts, such as those in our “signature” haemolysis set, are present in a plasma sample, their relative abundance is increased in the resulting sequencing library [19]. This is evidenced in a higher geometric mean of “signature” miRNAs relative to that of the other “background” miRNAs as demonstrated in Supplementary Figure S2. DraculR leverages this observation to evaluate and present evidence of haemolysis contamination.

**Figure 2:**
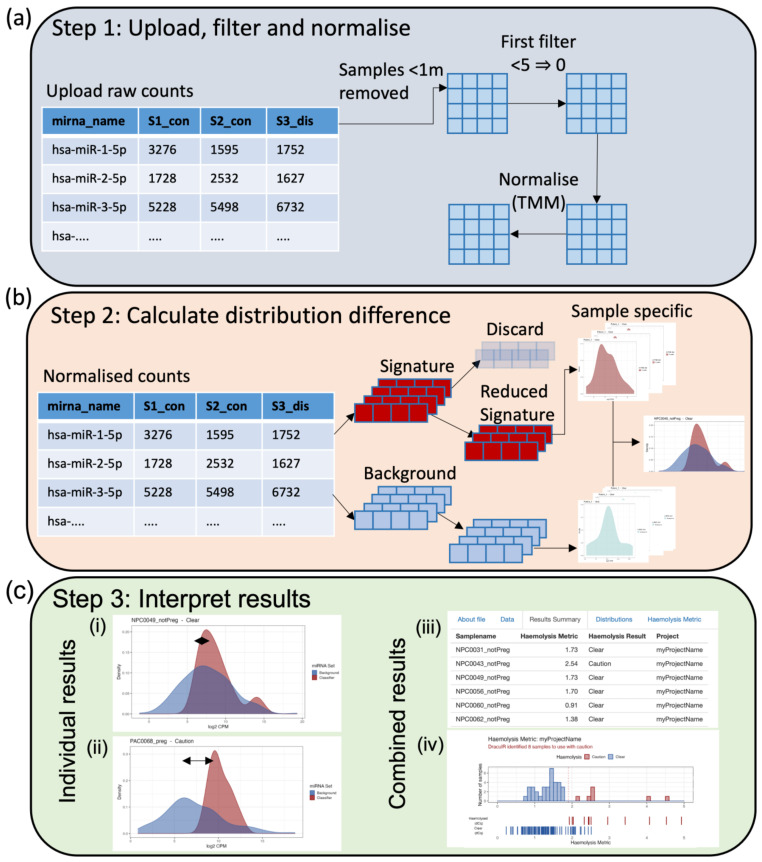
(**a**) Import a raw counts table generated by high-throughput miRNA sequencing of human plasma libraries. These data will be filtered according to user-specified requirements (*n* = number of samples in the smallest group of interest) and normalised using the Trimmed Mean of M (TMM) method [22]. (**b**) The distribution difference between the background and signature miRNA counts is calculated on an individual sample basis allowing the user to upload one to many samples as required. In the case of a priori knowledge of miRNA differentially abundant between a tested condition/control paradigm, the user may choose to reduce the signature miRNA such that they do not include miRNA of interest (recommended). (**c**) Graphical results in the form of a density plot of individual distributions (i, ii) and a histogram of combined distribution differences (iv) are provided along with a combined table of results (iii). The user is provided with both a metric describing the amount of haemolysis and, if appropriate, a recommendation of caution (iii).

DraculR can accept input as either raw or normalised miRNA expression data labelled with mature miRNA names in miRBase format (e.g., hsa-miR-106b-3p) [22] or will normalize raw data using the Trimmed Mean of M method (TMM) previously recommended [23]. The user has the ability to control features such as filtering for low expression (Figure 2a) as well as the option to refine the haemolysis signature set by discarding microRNAs (Figure 2b) including those with a priori knowledge of differential expressions in the comparison of interest. The purpose of removing miRNAs with a known association to the condition of interest is to help ensure any issues with haemolysis are not confounded with the research hypothesis. Note that samples with total miRNA read counts < 1 million are considered to be poorly sequenced and are removed for quality control.

DraculR then analyses and visualises the miRNA expression level distributions and, from these, calculates the Haemolysis Metric. Sample-specific and consolidated graphics of these including density plots, histograms, and tables are displayed (Figure 2c), including comparing the distributions of miRNA expression levels from the haemolysis signature set to that from background miRNAs. The sample-specific Haemolysis Metric for user-defined data is returned in tabular and graphical format for download and assessment, which helps the user decide on the level of haemolysis that may impact their analysis. The Haemolysis Metric values are evaluated against our recommended threshold of 1.9, selected to be comparable to the threshold used in the gold standard ΔCq (miR-23a-3p—miR-451a) method (details in Smith et al. [19]). Samples with Haemolysis Metric values ≥ 1.9 are interpreted as having levels of red-blood-cell-associated miRNAs consistent with haemolysis having occurred. Haemolysed samples are labelled with ‘Caution’ in the ‘Haemolysis Result’ column of the ‘Results Summary’ tab. Using the Haemolysis Metric, researchers/clinicians can assess their samples for evidence of haemolysis and obtain recommendations for their own individual samples as clear for use (‘Clear’) or use with caution (‘Caution’). Prior to use in any downstream analysis, we recommend removal, or at a minimum further investigation, of any samples that return a Haemolysis Metric above the threshold set here.

### 3.2. Public Data Example

To illustrate the utility of the application, we analysed four publicly available human plasma high-throughput sequencing miRNA datasets from the NCBI Gene Expression Omnibus database [24,25]: GSE153813, GSE118038, GSE105052, and GSE151341 [26,27,28]. These four analyses are available in the Supplementary Materials (Figures S3–S10)  and have also been used to demonstrate our method on the DraculR app through the “Public Data Example” tab. The signature set of miRNAs used to calculate the Haemolysis Metric initially comprises 20 miRNAs. Prior to calculating the Haemolysis Metric, any miRNAs whose differential expression correlates with the biology of the dataset should be excluded from both the background and signature sets. For the analysis of each of the public datasets, we identified and dropped any miRNAs from the signature set which were found either from the associated publication or a literature search to be differentially expressed between the conditions studied. In each example dataset, we were able to detect evidence of haemolysis in multiple samples (Table 1).

**Figure 3:**
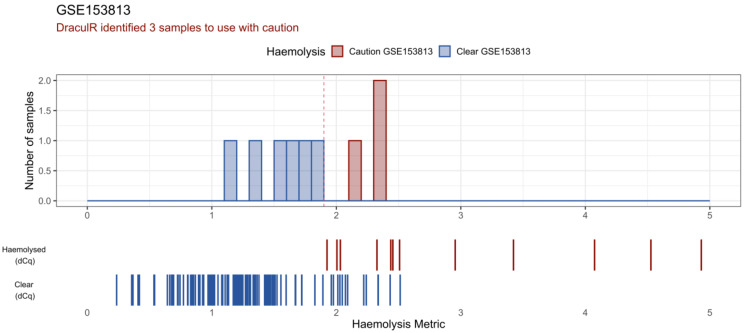
DraculR uses public data to illustrate the potential for unidentified haemolysis that could confound biomarker analysis. Here, miRNA expression data from NCBI GEO (GSE153813) was analysed and visualised. This screenshot shows the main DraculR visualisation of the data. The histogram represents the queried dataset where three samples were flagged to be used with caution (red) and six were classified as clear of haemolysis (blue). As a reference, the barcode-style plots below the histogram display the Haemolysis Metric values of samples from an example dataset validated using the ΔCq method (Smith et al.); red for those identified as haemolysed (ΔCq > 7), blue for those classified as ‘Clear’ of haemolysis (ΔCq < 7).

DraculR provides a visual representation of the results in the form of a histogram (Figure 3). In this histogram, the results from the user’s data are visualised in the context of a validated experiment from Smith et al. [19] where haemolysis was quantified via both the Haemolysis Metric and ΔCq (miR-23a-3p–miR-451a) methods. The user’s Haemolysis Metric values are visualised as solid colours (blue: ‘Clear’ or red: ‘Caution’) in the upper bar plot. For context, we provide Haemolysis Metric data from our previous, validated experiment as a barcode below. The upper row (Haemolysed (dCq)) Haemolysis Metric values are calculated from data where the original plasma sample returned a ΔCq (miR-23a-3p–miR-451a) value > 7. The lower row (Clear (dCq)) Haemolysis Metric values are calculated from data where the original plasma sample returned a ΔCq (miR-23a-3p–miR-451a) value < 7. Colour denotes the ΔCq (miR-23a-3p–miR-451a) value with red for those identified as haemolysed (ΔCq > 7) and blue for those classified as ‘Clear’ of haemolysis (ΔCq < 7). As reported in Smith et al. [19], we note that these data are best used for a general comparison only as pregnancy status may have affected the ΔCq results and therefore the concordance between the Haemolysis Metric and ΔCq.

## 4. Discussion

The abundance of cell-free miRNA has been measured in blood plasma and proposed as a source of novel, minimally invasive disease biomarkers [8,27,28]. However, an important but often overlooked factor is the potential for sample haemolysis during blood collection or sample preparation resulting in miRNA from lysed red blood cells contaminating the plasma sample [12,13,15]. When red-blood-cell-associated miRNA transcripts are retained and incorporated into the plasma-derived sequencing library, the relative abundance of these miRNAs is greater [19], and other miRNA expressions appear lower than in a pure plasma sample taken from the same individual. Using our previously reported Haemolysis Metric based on a signature set of haemolysis miRNAs, the user can assess their samples for evidence of haemolysis and obtain tailored recommendations as clear for use or use with caution. Using high-throughput sequencing data, DraculR is designed to visualise and analyse the distributions of miRNA expression levels for a haemolysis signature set compared to other (‘background’) miRNAs.

While research into the impact of haemolysis on miRNA quantification and normalisation continues, this is the first report of a publicly available method for the in silico identification of haemolysis in high-throughput sequencing data. Given that red blood cells are known miRNA repositories, without a robust method of haemolysis detection, measurements of miRNA abundance as disease biomarkers are limited [12] and there is a risk of false positive biomarker discovery as shown in [13,15]. These changes highlight the importance of understanding where data complexity originates.

One of the main strengths of DraculR over the q-PCR-based alternative approach is the robustness which comes from using 20 rather than a one miRNA for haemolysis detection. This in-built redundancy makes the method robust for cases where a case-control style analysis is undertaken and allows the user to use a priori knowledge of miRNA that is anticipated to be differentially abundant between groups to exclude these individual or multiple miRNAs from the Haemolysis Metric calculations. This gives greater confidence in haemolysis detection across a wide range of research questions.

DraculR is intended to be widely accessible so that noncomputational wet lab and clinical as well as bioinformatic researchers may identify potential haemolysis in a sample before proceeding further with downstream bioinformatics analyses. The application is light weight, easy to use, and reports a simple Haemolysis Metric and a clear recommendation regarding the downstream analysis of each of the samples being considered. This simple, but important, quality check is rarely reported in the literature relating to miRNAs in human plasma or serum, and its implementation will improve the confidence and quality of research in this field.

The probabilistic quantification of contamination risk is not possible based on the dataset used here, and future work drawing on the methods used by Shah et al., (2016) [29] may include serial dilution and the miRNA quantification of haemolysed plasma samples to validate and further refine the method. DraculR adds value to the growing resource of public data shared by plasma researchers by enabling an in silico analysis of haemolysis confounding post sequencing. The detection of haemolysis using our Haemolysis Metric enables the user to identify and potentially discard low quality samples which are otherwise not recognised as being affected by haemolysis. This provides an additional quality metric and the subsequent increased confidence in the use of miRNA high-throughput sequencing expression data for which no haemolysis information is available. Further details and examples are provided in the Supplementary Materials.

## 5. Conclusions

We developed DraculR, a Shiny/R web-based application that allows users to detect and address the issue of haemolysis in plasma miRNA high-throughput sequencing data. This software addresses the need for quality control where, either through the use of public data, exhaustion of the sample, or exhaustion of funds, it is not possible to assess haemolysis using one of the current gold standard approaches (being delta quantification cycle (ΔCq) values for miR-23a-3p–miR-451a, or Spectrophotometry for haemoglobin estimation). It is available online at https://mxhp75.shinyapps.io/DraculR or from our GitHub repository for local deployment (https://github.com/mxhp75/DraculR).

## Figures and Tables

**Table 1:** Publicly available human plasma miRNA expression data were assessed for haemolysis using the DraculR method, identifying multiple samples in each dataset that had sufficient evidence of haemolysis to recommend caution in their use. No haemolysis information was included with the original dataset.

Dataset	Experimental Context	Total Samples	Caution	Differentially Abundant miRNA	Publication
GSE153813	Case: Control Profile miRNA expression at each stage of menstrual cycle; endometriosis	9	3	NA	NA
GSE105052	Case: Control Friedreich’s ataxia	42	3	hsa-miR-128-3p, hsa-miR-625-3p, hsa-miR-130b-5p, hsa-miR-151a-5p, hsa-miR-330-3p, hsa-miR-323a-3p, hsa-miR-142-3p	[26]
GSE151341	Case: Control Early radiographic knee osteoarthritis biomarker	91	4	hsa-miR-335-3p, hsa-miR-199a-5p, hsa-miR-671-3p, hsa-miR-1260b, hsa-miR-191-3p, hsa-miR-191-5p +, hsa-miR-335-5p, hsa-miR-543	[27]
GSE118038	Case: Control Prostate cancer biomarker	70	32	hsa-miR-4732-3p, hsa-let-7a, hsa-miR-26b-5p, hsa-miR-98-5p, hsa-miR-30c-5p *, hsa-miR-21-5p, hsa-miR-191-5p +	[28]

* Haemolysis-Metric-associated miRNA, DE reported in associated publication. + Haemolysis-Metric-associated miRNA, DE reported in wider literature search.

## Data Availability

DraculR and its tutorial are freely available from (https://mxhp75.shinyapps.io/DraculR/). Code for running locally is available from (https://github.com/mxhp75/DraculR) under the GNU General Public License v3.0.

## Supplementary Materials

The following supporting information can be downloaded at https://www.mdpi.com/article/10.3390/genes14020448/s1: Figure S1: DraculR provides a simple GUI for data upload and manipulation. First, the user selects a local file for upload (a) and changes the project title to be used on tables and figures (b). Prior to the algorithm running, the user then selects the minimum number of samples per group for filtering (c) and selects any miRNA which are known to be differentially abundant between the user groups of interest (d) to be dropped from the Haemolysis Metric calculation. Figure S2: In the first example (a) the sample is classified as ‘Clear’, indicating no evidence for haemolysis. The distance between the geometric mean of background and signature set miRNA is small. In the second example (b) the sample is classified as ‘Caution’, indicating that we found evidence suggestive of haemolysis. The geometric mean of background and signature set miRNA is further apart than that expected where no haemolysis is present. Figure S3: Sequencing read depth for GSE153813. Dashed line represents one million reads. Figure S4: DraculR identified 3 samples from GSE153813 to be used with caution in downstream analysis. Figure S5: Sequencing read depth for GSE118038. Dashed line represents one million reads. Figure S6: DraculR identified 32 samples from GSE118038 to be used with caution in downstream analysis. Figure S7: Sequencing read depth for GSE105052. Dashed line represents one million reads. Figure S8: DraculR identified three samples from GSE105052 to be used with caution in downstream analysis. Figure S9: Sequencing read depth for GSE151341. Dashed line represents one million reads. Figure S10: DraculR identified four samples from GSE105052 to be used with caution in downstream analysis.
