# Supplementary material for: DraculR: A Web-Based Application for In Silico Haemolysis Detection in High-Throughput microRNA Sequencing Data
Source: Genes (Basel). 2023 Feb 9;14(2):448. doi: 10.3390/genes14020448 (PMC9957079; doi:10.3390/genes14020448)
Supplement: Supplementary file 1 [file genes-14-00448-s001.zip › genes-2140785-supplementary.pdf]

Supplementary Data

The Haemolysis metric described here [19] and implemented in the DraculR Shiny/R web-based application, performs an *in silico* quality assessment to detect evidence of haemolysis contamination in the original plasma specimen, assigning each small RNA sequencing dataset into one of two categories. The classification of 'Clear' or 'Caution' alert the user to potential quality control issues in specimen.

Application

DraculR provides a simple graphical user interface (GUI) that allows the user to upload small RNA sequencing data in the form of a raw counts table with names in mature miRNA format (e.g. hsa-miR-106b-3p) [22] (Figure S1a). The interface provides the option to personalise table and figure titles (Figure S1b), set filtering options (Figure S1c) and remove miRNA known to be differentially abundant between the user groups of interest from the calculation of the Haemolysis Metric (Figure S1d). This final step ensures the calculation for haemolysis is not confounded in the event that one or more of the miRNA signature set is known to differ between groups.

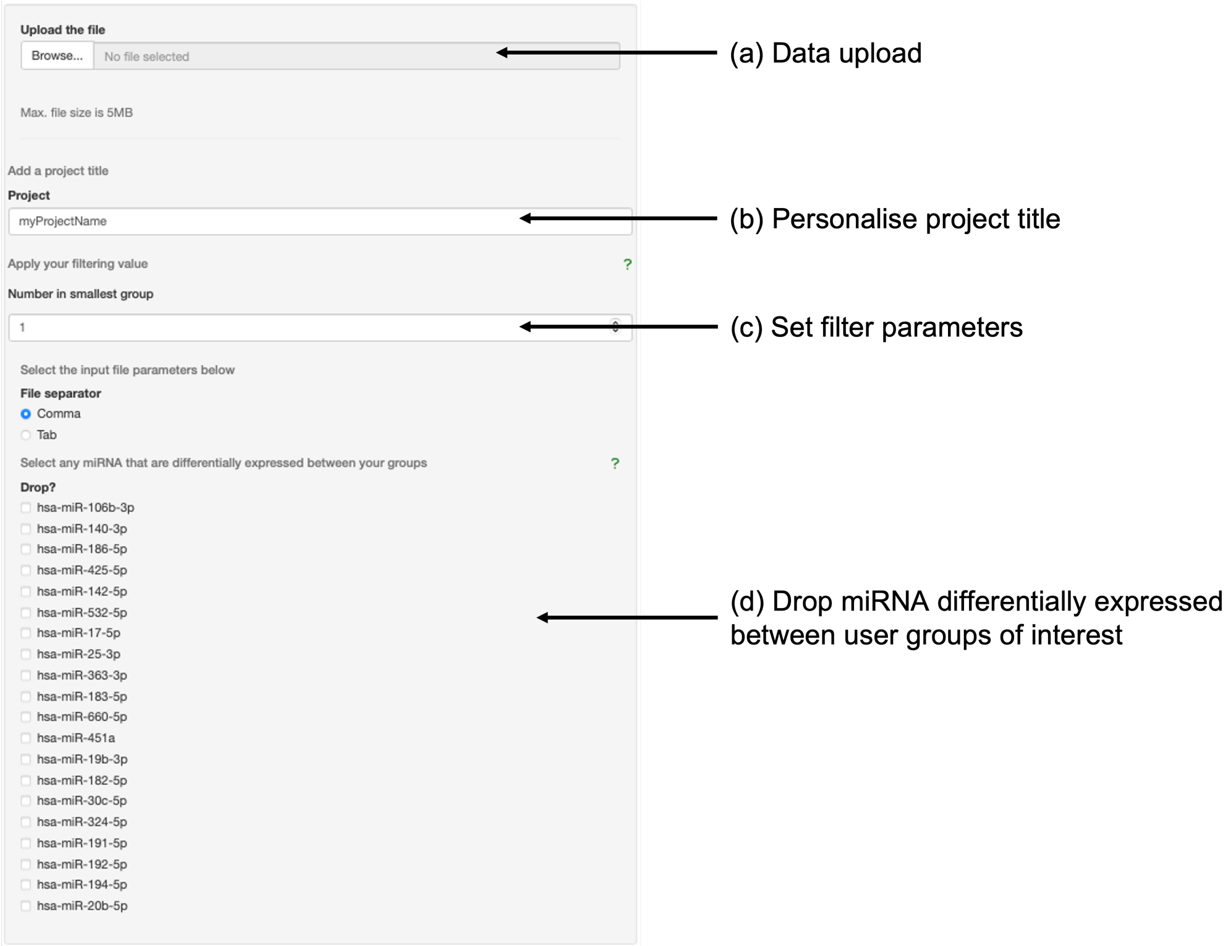

Figure S1. DraculR provides a simple GUI for data upload and manipulation. First, the user selects a local file for upload (a) and changes the project title to be used on tables and figures (b). Prior to the algorithm running, the user then selects the minimum number of samples per group for filtering (c) and selects any miRNA which are known to be differentially abundant between the user groups of interest (d) to be dropped from the Haemolysis metric calculation.

In Figure S2 we present an example distribution illustrative of a dataset classified as Clear (Figure S2a) and as one as Caution (Figure S2b). In the first example (Figure S2a), the distance between the geometric mean of the background miRNA (light blue) compared to that of the signature set miRNA (scarlet) is very small whereas in the second example (Figure S2b) the distance between the geometric mean of the background miRNA compared to that of the signature set miRNA is larger. Furthermore, this difference is greater than the Haemolysis Metric threshold of 1.9, established in Smith *et al.* [19], and thus the sample has been classified as 'Caution'. In Figure S2b the signature set distribution suggests these microRNAs are over-represented relative to Figure S2a. This

suggests that RBC-associated miRNA have been added to the pool of miRNA isolated in the plasma. In the haemolysed example (Figure S2b), we would recommend removing the sample data from further analysis. However, where a decision is made to retain samples, the issue of haemolysis should be noted and may be a limitation to inference.

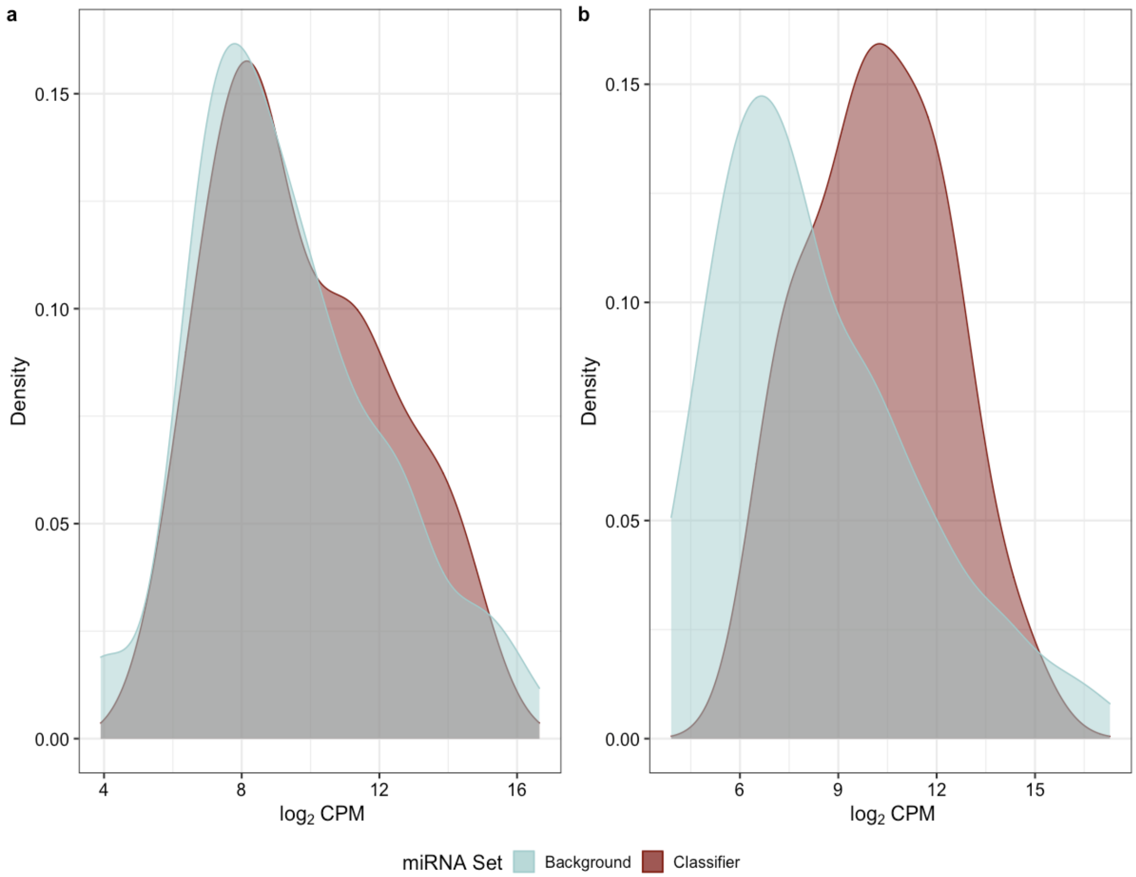

Figure S2. In the first example (a) the sample is classified as ‘Clear’ indicating no evidence for haemolysis. The distance between the geometric mean of background and signature set miRNA is small. In the second example (b) the sample is classified as ‘Caution’ indicating that we found evidence suggestive of haemolysis. The geometric mean of background and signature set miRNA is further apart than that expected where no haemolysis is present.

Public data example

To illustrate the utility of the application, we used DraculR to analyse four publicly available human plasma high throughput sequencing miRNA datasets from NCBI GEO [24-25]: GSE153813, GSE118038, GSE105052, GSE151341 [26-28].

In GSE153813, RNA libraries were prepared using human plasma and subjected to high throughput sequencing. The aim of the study was to profile miRNA expression across the menstrual cycle in the context of endometriosis, a disorder characterised by the growth of uterine tissue outside of the uterus. Given the nature of the experiment, all participants were female. Samples were grouped by health status, Endometriosis (n = 6) and Healthy (n = 3). An average of ~2.2 million reads (Figure S3) were sequenced per sample (range 0.96 to 3.8 million reads). All libraries were retained for analysis using DraculR, which identified three samples to be used with caution in downstream analysis (Figure S4).

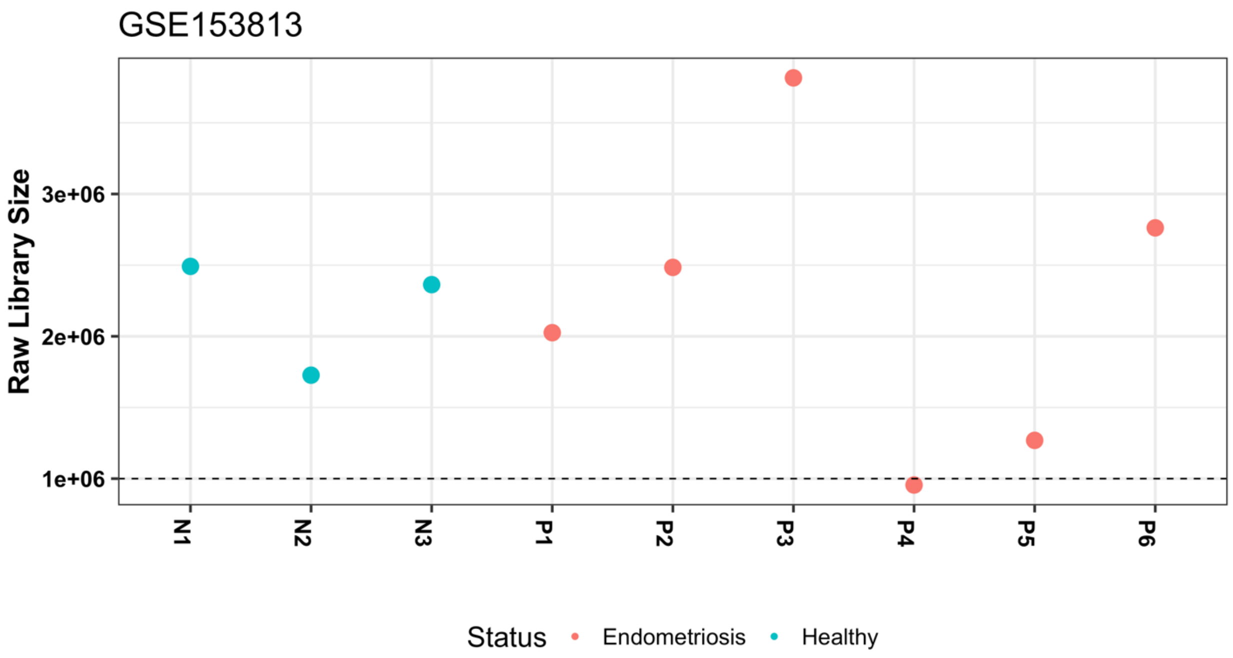

Figure S3. Sequencing read depth for GSE153813. Dashed line represents one million reads.

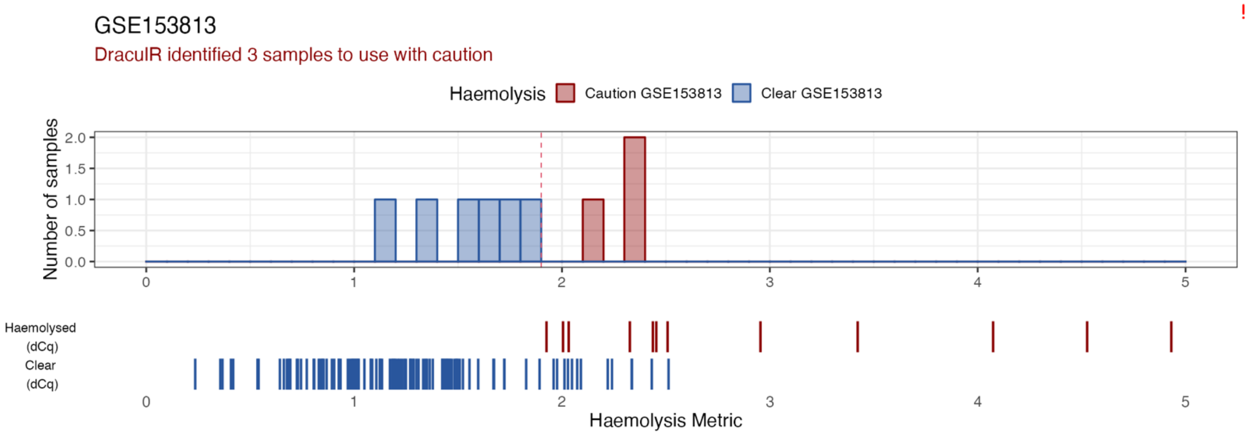

Figure S4. DraculR identified 3 samples from GSE153813 to be used with caution in downstream analysis.

In GSE118038, RNA libraries were prepared using human plasma and sequenced to generate expression profiles. The aim of this study was to explore the biomarker potential of plasma derived miRNAs in the context of prostate cancer diagnosis [28]. Given the nature of the experiment, all participants were male. Samples were grouped by health status, Prostate Cancer (n = 33) and Healthy (n = 37). An average of ~0.7 million reads (Figure S5) were sequenced per sample (range 0.21 to 1.91 million reads). This study identified six miRNA that were differentially abundant between healthy individuals and those diagnosed with prostate cancer (Table 1 of the main paper). Of these, one, miR-30c-5p, forms part of the 20 miRNA signature set and was dropped from the Haemolysis metric calculation using the option shown in Figure S1b. All libraries were retained for analysis using DraculR, which identified 32 samples to be used with caution in downstream analysis (Figure S6).

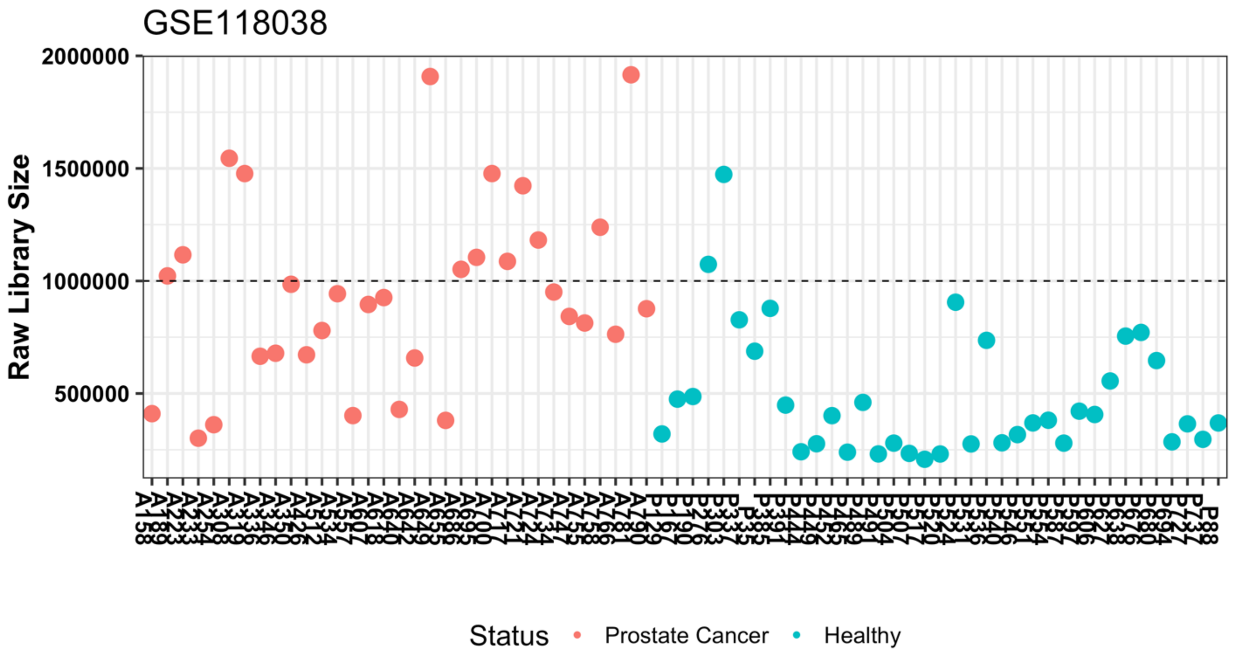

Figure S5. Sequencing read depth for GSE118038. Dashed line represents one million reads.

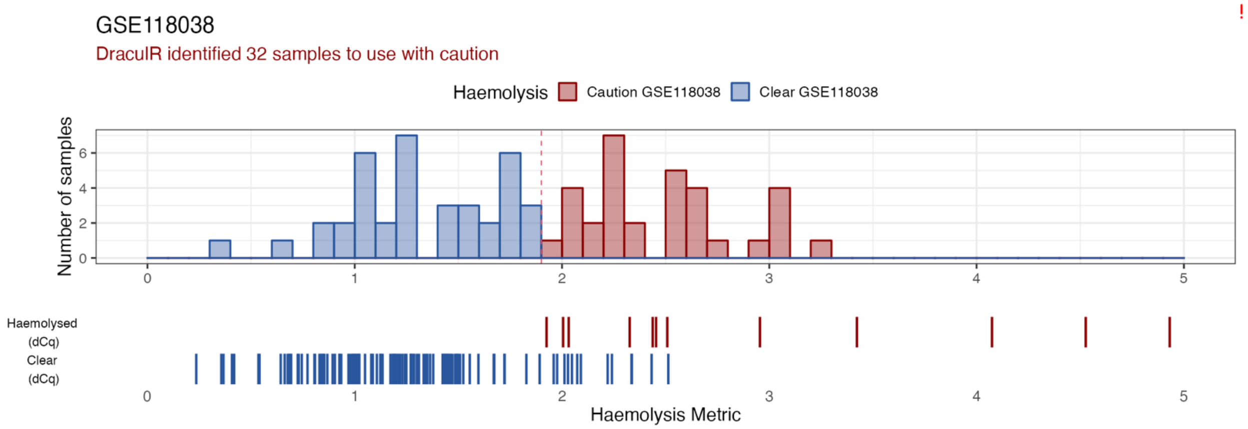

Figure S6. DraculR identified 32 samples from GSE118038 to be used with caution in downstream analysis.

In GSE105052, RNA libraries were prepared using human plasma and sequenced to generate miRNA expression profiles. The aim of this study was to explore the biomarker potential of plasma derived miRNAs in the context of Friedreich's ataxia [26]. Samples were grouped by health status, Friedreich's ataxia (n = 25) and Healthy (n = 17). An average of ~0.13 million reads (Figure S7) were sequenced per sample (range 0.0014 to 0.69 million reads). This study identified seven miRNA that were differentially abundant between healthy individuals and those diagnosed with Friedreich's ataxia (Table 1 of the main paper), none of which is part of the 20 miRNA signature set used in the Haemolysis metric calculation. Noting but in spite of the extremely low sequencing depth, we analysed the data using DraculR which identified three samples to be used with caution in downstream analysis (Figure S8).

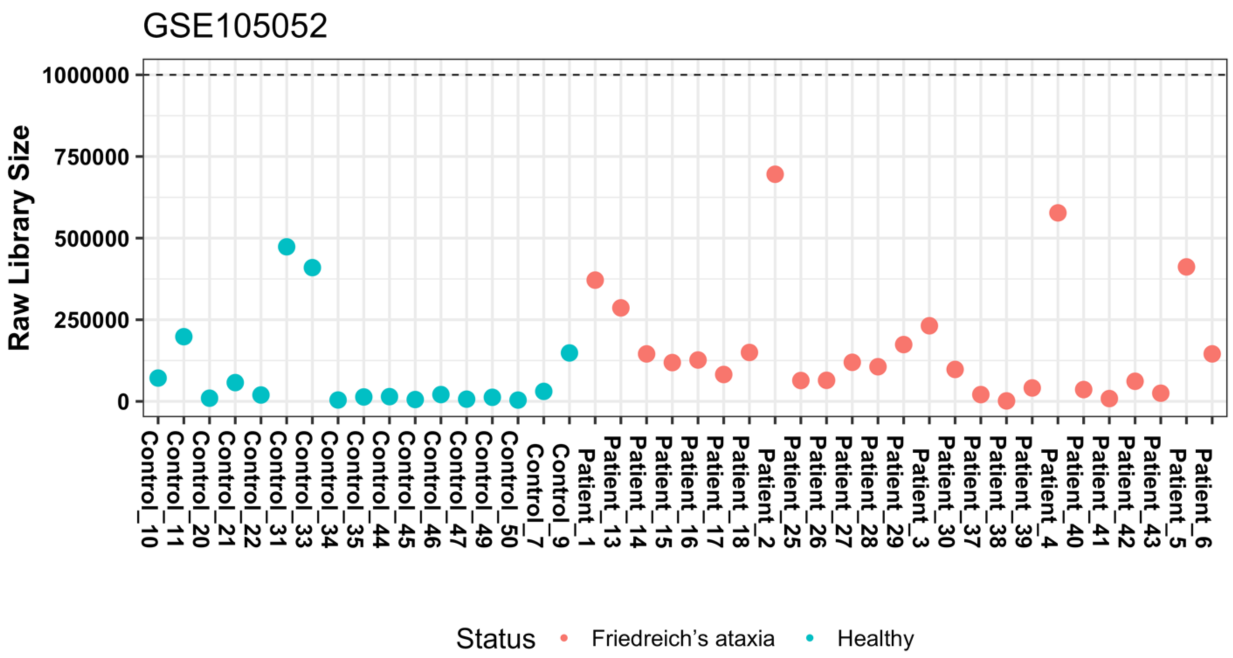

Figure S7. Sequencing read depth for GSE105052. Dashed line represents one million reads.

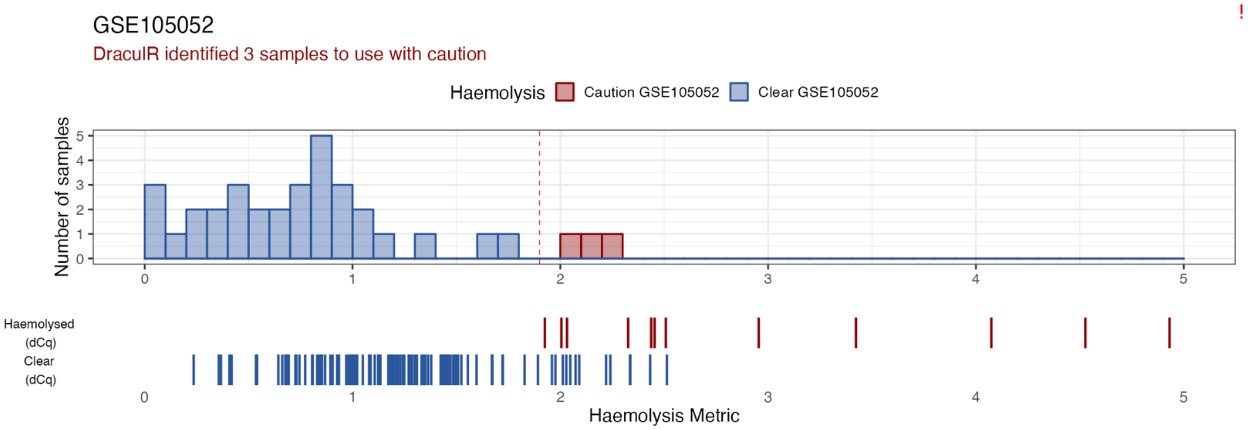

Figure S8. DraculR identified three samples from GSE105052 to be used with caution in downstream analysis.

In GSE151341, RNA libraries were prepared using human plasma and sequenced to generate expression profiles [27]. The aim of the study was to sequence plasma miRNAs from patients with radiographic knee osteoarthritis to identify unique miRNA signatures in each of two disease states. Samples were grouped by disease status, early [Kellgren-Lawrence grade 0 or 1 (n=41)] or late [Kellgren-Lawrence grade 3 or 4 (n=50)] symptomatic radiographic knee osteoarthritis. An average of ~2 million reads (Figure S9) was sequenced per sample (range 0.39 to 6.09 million reads). This study

identified seven miRNA that were differentially abundant between individuals diagnosed with early or late radiographic knee osteoarthritis (Table 1 of the main paper), none of which was part of the 20 miRNA signature set used in the Haemolysis metric calculation. All libraries were retained for analysis using DraculR which identified three samples to be used with caution in downstream analysis (Figure S10).

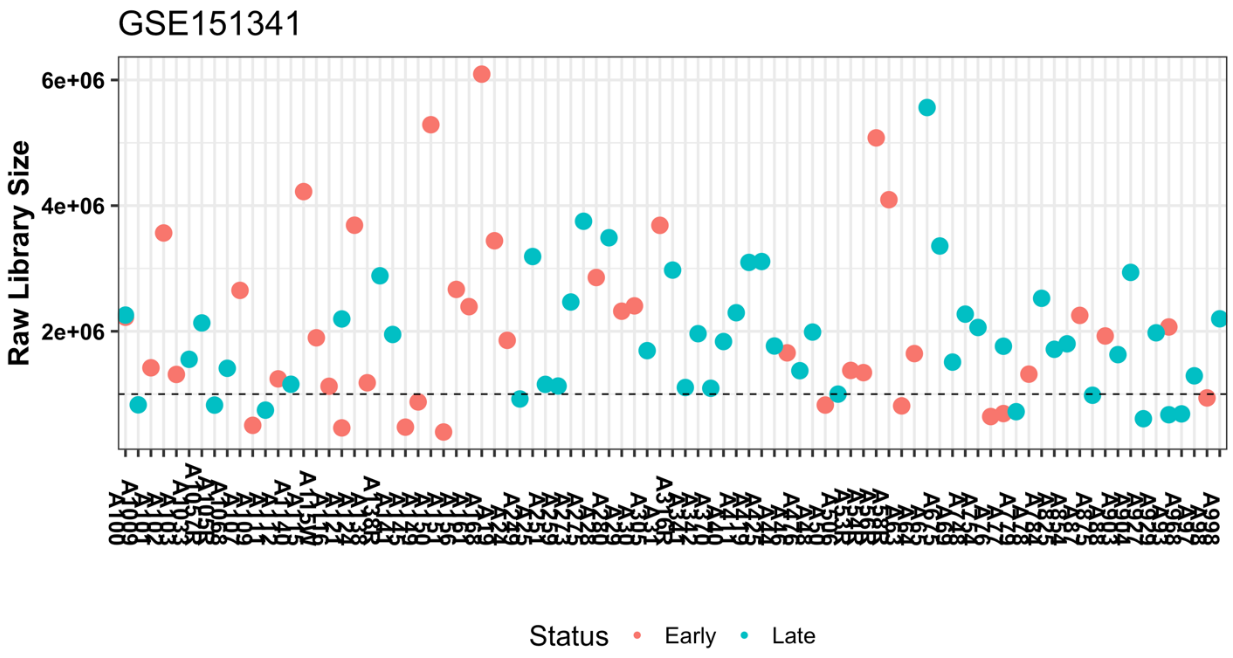

Figure S9. Sequencing read depth for GSE151341. Dashed line represents one million reads.

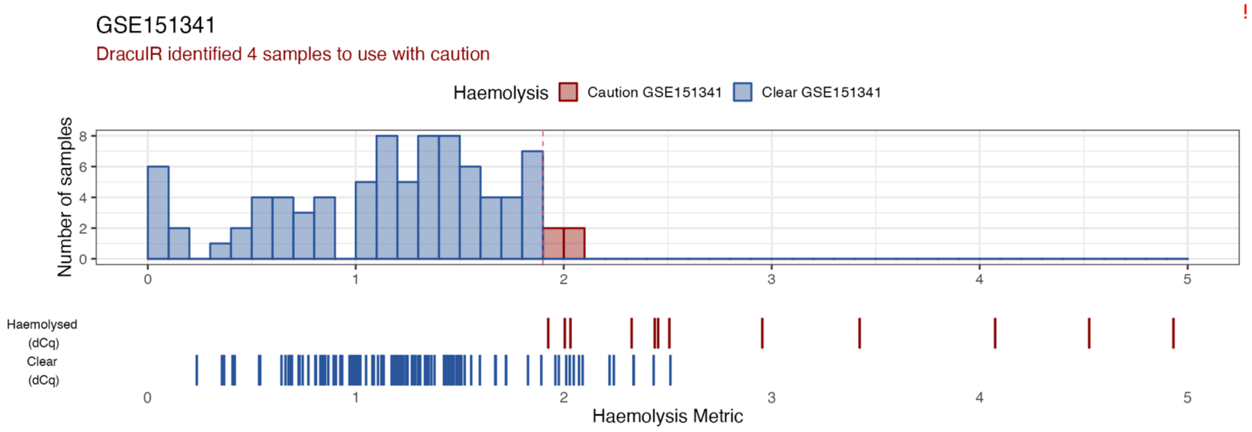

Figure S10. DraculR identified four samples from GSE105052 to be used with caution in downstream analysis.

Detailed information regarding each of the public data sets can be found in Table 1 of the main paper.
